# Operating room professionals’ attitudes towards patient safety and the influencing factors

**DOI:** 10.12669/pjms.335.13615

**Published:** 2017

**Authors:** Pinar Ongun, Seyda Seren Intepeler

**Affiliations:** 1 Pinar Ongun, M.Sc.Research Assistant, Istanbul Aydin University Emergency and First Aid Program, Istanbul, Turkey; 2 Seyda Seren Intepeler, BSN, PhD.Associate Professor, Department of Nursing Management, Nursing Faculty, Dokuz Eylul University, Izmir, Turkey

**Keywords:** Attitude of Health Personnel, Operating room, Patient safety

## Abstract

**Objective:**

To determine operating room professionals’attitudes towards patient safety and the influencing factors.

**Methods:**

This study was conducted in research hospitals in Izmir, Turkey using descriptive, cross-sectional and correlation research designs. The sample of this study consisted of 477 individuals including nurses, physicians and anesthesia technicians. Data were collected using the Sociodemographic and Working Characteristics Form and the Safety Attitudes Questionnaire. Descriptive statistics method, and Pearson’s correlation and the multiple regression models were used for data analysis.

**Results:**

Operating room professionals’ attitudes towards patient safety were at moderate levels. Regarding the influencing factors, team cooperation obtained the highest score, whereas stress recognition obtained the lowest score. As a result of the regression analysis, age, male gender and receiving patient safety training explains 15.4% of the professionals’ safety attitudes.

**Conclusions:**

Receiving patient safety training was found to be the most important variable of all.

## INTRODUCTION

The most significant developments related to patient safety began to take shape following the publication of the “To Err is Human” report by the Institute of Medicine and the dramatic results on the patient safety have been obtained.^1^,^2^ The fact that medical errors are the fifth leading cause of death emphasizes the importance of the issue.^2^

The higher the number of operations is, the higher the number of risks and adverse events related to patient safety gets.^2^,^3^ Also, the high number of risk factors in operating rooms (OR) threatens the patient safety. Operating room settings are marked by an increased use of technological devices and the presence of different occupational groups, who work long, erratic hours and cope with many problems.^2^,^3^ Due to breakdowns in teamwork and communication, these problems can potentially result in a higher number of errors.^4^-^9^ In turn, these errors that are committed in the OR can negatively impact patients, families, caregivers and the institution itself.

Medical errors such as leaving sponges in the operative field, wrong-organ transplantation, incorrect blood transfusion or wrong site operations caused by interprofessional communication and cooperation problems are frequently encountered in OR.^5^-^8^,^10^,^11^

The establishment of a suitable patient safety culture is associated with the expectations and actions of directors, institutional learning, teamwork, interprofessional open communication, appropriate feedback, the support of hospital administration and personnel education.^12^ It has been shown that medical errors decrease when this patient safety culture is adopted by all employees of the institution. Since OR are one of the settings where medical errors are mostly observed, the data obtained as a result of determining operating room professionals’ attitudes towards patient safety will provide benefits in terms of identifying risk factors for patient safety, raising awareness in institutions and serving as an information source for operating room professionals and directors to conduct a preventive action.

The current study aimed to determine operating room professionals’ attitudes towards patient safety and the factors influencing the attitudes.

## METHODS

This study was conducted using descriptive, cross sectional and correlation research designs from March, 2014–June, 2015 at all university training and research hospitals in Izmir, Turkey. The study population included the professionals working in the OR of these hospitals (N: 1581). Improbable sampling method was used to determine the sample of the study. The sample group consisted of nurses, physicians and anesthesia technicians who had agreed to participate in the study and had been working in OR for at least six months (n=477), except for the ones who were either on personal leave or on sick leave. The period of six months was regarded to be adequate for perceiving the organization culture and function. The dependent variable of the study was operating room professionals’ attitudes towards patient safety, while the independent variables included operating room professionals’ages, genders, educational levels, working positions and average working hours, their experiences in their respective profession, in-service training practices in the institution and their participation in these training practices and their educational levels in terms of patient safety.

### Ethical Considerations

Permissions were obtained from the institutions where the study was conducted, and ethical approval was obtained from the Ethics Committee of Non-interventional Clinical Studies (decision date: 18.12.2014, decision no: 2014/37-35). The aim of the study was explained to all study participants through the Informed Voluntary Consent Form, their consents were received and the data were collected according to the principle of voluntary participation.

Data were collected using the Sociodemographic and Working Characteristics Form and the Safety Attitudes Questionnaire (SAQ) (Operating room version), which were prepared through the literature review.^8^,^13^-^15^ The questionnaire, which was developed by Sexton et al. (2006)^15^ and adapted into Turkish by Onler (2010)^13^, included 58 items and the following six sub-scales: teamwork cooperation, job satisfaction, perceptions of management, safety climate, working conditions, and stress recognition. A five point Likert-type scale was used in the questionnaire. The higher the scores obtained from the questionnaire were, the more positive attitudes its sub-scales indicated. The reliability coefficient was 0.90 for the original questionnaire and 0.92 for its Turkish adaptation. In the current study, this coefficient was found to be 0.91.^13^,^15^ The researcher delivered the questionnaires to the physicians, nurses and anesthesia technicians and collected them back in an anonymous manner to ensure privacy.

### Data Analysis

All data were analyzed using the SPSS 21.00 program. Descriptive statistics were administered to interpret the sociodemographic data. The correlation among age, gender, profession, duration of employment, weekly hours and participation in operating room professionals’ patient safety training and the mean scores of their safety attitudes was analyzed using Pearson’s correlation model. The factors which influenced their safety attitude scores were analyzed using the multiple regression models. The statistical significance level was regarded as p<0.05.

## RESULTS

The mean age of the study sample was 35.16 (SD=8.68). Of them, 41.9% were between the ages of 30 and 39, 51.4% were male. Based on their educational levels, 29.1% were medical specialists, 35.4% were research assistants and 24.5% were nurses.

Furthermore, 57% worked more than 40 hours in a week and their duration of employment was 11.16 (SD=9.05) years on average. Besides, 73.3% reported that they could not take a rest from their working hours. It was reported by 67.7% of the participants that no orientation program had been administered when they first started to work in the OR, and 58.5% stated that no regular in-service training was provided in the operating room. Of those who participated in an in-service program, 75.4% reported that the needs of the participants were taken into consideration in determining the program. Of the group, only 46.5% had participated in a patient safety training program. Of them, 91.4% had participated in a patient safety in-service training program.

The analysis of the professionals’ safety attitudes found the total means score of the scale to be 49.99±12.24. This mean scores are summarized in Table-I. Professionals’ attitudes were at a moderate level, and only the attitudes related to the stress level were at a low level.

**Table-I:** Mean score of SAQ and sub-scales.

*SAQ and Sub-Scales*	*Mean Score*	*SD*
SAQ	49,99	12,24
Teamwork Cooperation	59,27	13,91
Job Satisfaction	57,74	22,01
Safety Climate	53,35	13,97
Working Conditions	51,57	22,45
Perceptions of Management	44,59	22,47
Stress Recognition	33,94	15,44

Correlations among the safety attitude mean score, the safety attitude sub-scales and the influencing factors are shown in Table-II. The safety attitude mean score was found to have a strong, positive correlation with its teamwork cooperation (r=0.829), job satisfaction (r=0.803), the support of management (r=0.838), safety climate (r=0.885) and working conditions (r=0.749) sub-scales, and it had a weak, positive correlation with stress level.

**Table-II:** The correlation between the safety attitudes mean score, the safety attitudes sub dimensions and the factors affecting.

	*SAQ*	*Teamwork climate*	*Job Satisfaction*	*Perceptions of Management*	*Safety climate*	*Working Conditions*	*Stress Recognition*	*Age*	*Gender*	*Job*	*Working years in the profession*	*Weekly working hours*	*Participate in patient safety training*
SAQ	1												
Teamwork climate	.829	1											
Job Satisfaction	.803	.688	1										
Perceptions of Management	.838	.641	.746	1									
Safety climate	.885	.722	.641	.688	1								
Working Conditions	.749	.641	.611	.717	.638	1							
Stress Recognition	.363	.028	.104	.103	.154	.043	1						
Age	.284	.225	.296	.240	.228	.247	.091	1					
Gender	.127	.064	.201	.117	.051	.021	.127	.066	1				
Job	-.078	-.121	-.134	-.088	-.053	-.030	.061	-.034	-.146	1			
Working years in the profession	.222	.193	.186	.172	.187	.226	.071	.831	-.107	.169	1		
Weekly working hours	-.195	-.136	-.112	-.141	-.145	-.192	-.162	-391	.217	.-357	-.531	1	
Participate in patient safety training	-.221	-.153	-.098	-.203	-.199	-.208	-.125	-0.40	.185	-.112	-.115	.208	1

The factors that influence professional`s attitudes towards patient safety according to the multiple regression analysis are shown in Table-III. A multiple regression analysis was used to identify the factors that affect professionals’ attitudes towards patient safety. The variables included in the model were determined using correlation analysis, whereby it was concluded that the specified variables explained 15% of the model. The most important variables were found to be age, participation in a patient safety training program, male gender and nursing profession (p<0.05)

**Table-III:** The determination of factors affecting the attitudes of professionals towards patient safety according to multiple regression analysis.

*Variable*	*B*	*Standard error*	*Standard beta*	*t*	*p*	*Tolerance*	*VIF*
Constant	41.502	3.177	-	13.065	0.000		
Age	0.361	.061	0.256	5.921	0.000	0.568	1.761
Participate in patient safety training	-5.462	1.109	-0.223	-4.923	0.000	0.961	1.041
Gender (Male)	4.620	1.274	0.189	3.628	0.000	0.548	1.823
Job (Nurse)	2.894	1.623	0.102	1.783	0.075	0.884	1.132
Educational status	0.242	0.354	0.038	0.683	0.495	0.879	1.138
Specialist	-0.796	1.664	-0.022	-0.478	0.633	0.664	1.506

R: 0.393, R²: 0.154, F: 14.278, p: 0.000, Durbin Watson: 1.782 (1.5-2.5).

## DISCUSSION

Similar to the other studies, the current study indicated that operating room professionals’ attitudes towards patient safety were found to be at the moderate level.^13^,^15^ Operating room professionals’ strongest positive attitudes among all sub-scales of the SAQ were found to be in the area of teamwork cooperation. Other studies have also shown teamwork cooperation scores to be similar to those found in the current study.^10^,^15^ High scores in teamwork among professionals play an important role in ensuring patient safety and creating a positive culture.^2^,^16^,^17^ Moreover, the establishment of effective interprofessional teamwork will support the development of a positive safety culture, and in turn it will increase the level of positive attitudes towards patient safety.

Stress recognition obtained the lowest score among the sub-scales of the operating room professionals’ SAQ. However, there are some studies in which the stress recognition score was found to be high.^13^,^14^ A study conducted in an operating room with the participation of surgeons reported that teamwork was one of the most stressful situations in OR.^18^ According to another study which examined the effect of job satisfaction in operation rooms on patient safety, operating room professionals’ high levels of stress affected job satisfaction and patient safety.^19^ The low stress level observed in the current study can be associated with the positive perception of teamwork in OR. It was proved that interprofessional teamwork had an effect on professionals’ stress levels.^7^,^18^ Low stress levels in OR is believed to affect the patient safety culture positively.

In the current study, a weak, positive correlation was found among safety attitudes’ mean score, age and participation in patient safety training programs. A low-level positive correlation was found between safety attitude and age, which were the sub-scales concerning job satisfaction. In other studies, a positively increasing correlation was found between receiving patient safety training and patient safety.^13^,^16^ Similarly, the patient safety attitudes of professionals whose job satisfaction level increased were found to be positive.^13^,^19^ It can also be stated that the older the professionals who work in an institution get, the higher the level of sense of belonging and loyalty they feel for the institution becomes. This case also leads to the increase in professionals’ job satisfaction level. An increase in job satisfaction level helps to reduce stress in working environment, therefore facilitates greater efficiency in teamwork and improves professionals’ safety attitudes.

Receiving patient safety training is one of the factors which influences and is crucial for safety attitude. It was found to be necessary in several other studies.^13^,^16^,^20^ The importance of providing patient safety training for professionals to create and maintain a culture concerning patient safety has been emphasized. It is thought to have an important effect on ensuring positive safety attitudes.

## CONCLUSION

As a result of this study, it is a huge responsibility of hospital administrators, unit supervisors and other professionals to create the most efficient and effective setting for a patient safety culture and maintain this positive setting. Administrators should ensure that the working hours of professionals comply with the law, plan their shifts in such a way to provide them with adequate time for rest; also provide them with an orientation program before they begin to work in OR, maintain in-service training programs on patient safety in accordance with the opinions of professionals to ensure their continuous development, provide support for incorporating the patient safety culture in the institution and maintaining its continuity. Researchers, on the other hand, should conduct semi-experimental studies to examine and improve the administrative support within the scope of improving operating room professionals’ attitudes towards patient safety. It is important to obtain generalizable results from the studies to be conducted in different cities in order to bring the patient safety in Turkey to the optimum level.
